# Kruppel-family zinc finger proteins as emerging epigenetic biomarkers in head and neck squamous cell carcinoma

**DOI:** 10.1186/s40463-023-00640-x

**Published:** 2023-05-30

**Authors:** Patrick Pearson, Kendra Smith, Nilita Sood, Elizabeth Chia, Alicia Follett, Michael B. Prystowsky, Simon Kirby, Thomas J. Belbin

**Affiliations:** 1 Division of Biomedical Sciences, Faculty of Medicine, Memorial University of Newfoundland, St. John’s, NL A1B 3V6 Canada; 2 Discipline of Oncology, Faculty of Medicine, Memorial University of Newfoundland, St. John’s, NL A1B 3V6 Canada; 3 Discipline of Laboratory Medicine, Faculty of Medicine, Memorial University of Newfoundland, St. John’s, NL A1B 3V6 Canada; 4 Department of Pathology, Albert Einstein College of Medicine, 1300 Morris Park Avenue, Bronx, NY 10461 USA

**Keywords:** ZNF, Oral cavity, Squamous, Carcinoma

## Abstract

**Background:**

Krüppel-type zinc finger protein genes located on chromosome 19q13 are aberrantly hypermethylated with high frequency in all anatomic sub-sites of head and neck cancers as well as other epithelial tumours resulting in decreased expression.

**Methods:**

We examined prognostic significance of ZNF154 and ZNF132 expression and DNA methylation in independent patient cohort of about 500 head and neck cancer patients in the Cancer Genome Atlas (TCGA). We also overexpressed these genes in HEK-293 cells, as well as the oral cancer cell line UM-SCC-1.

**Results:**

In 20 patients from the TCGA cohort of HNSCC patients where ZNF154 and ZNF132 DNA methylation and RNA expression could be compared in tumor and adjacent normal tissue, there was increased DNA methylation and decreased expression of both ZNF154 and ZNF132 in primary tumours. Low ZNF154 and low ZNF132 expression were associated with shorter overall survival in both head and neck squamous cell carcinoma (HNSCC) and lung adenocarcinoma (LUAC patients). While expression of these proteins in HEK-293 cells produced full-length protein, only truncated copies could be expressed in head and neck cancer cells (UM-SCC-1). The truncated version of ZNF154 protein increased doubling time and reduced cell migration in UM-SCC-1 cancer cells.

**Conclusions:**

Both ZNF132 and ZNF154 represent novel clinically significant biomarkers in head and neck cancer with potential tumour suppressive properties. Future studies will address the underlying molecular mechanisms by which ZNF154 expression in HNSCC contributes to the control of cell growth and migration.

**Graphical abstract:**

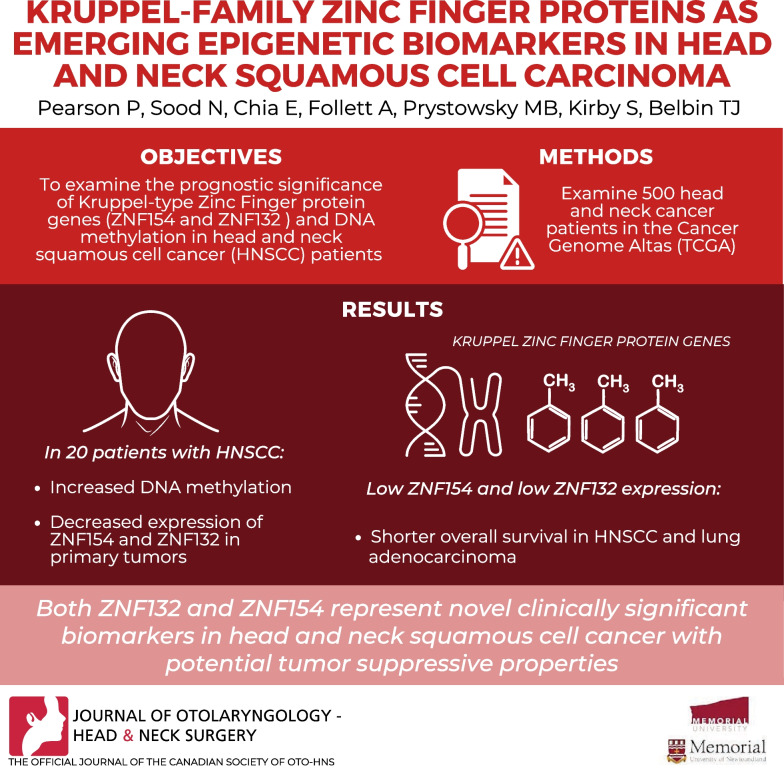

**Supplementary Information:**

The online version contains supplementary material available at 10.1186/s40463-023-00640-x.

## Introduction

Head and neck squamous cell carcinomas (HNSCCs) rank among the 10 most common malignancies in men and women worldwide. All have in common an etiological association with tobacco and/or alcohol exposure [1]. Overall, 5-year survival rates are approximately 50%, but there is substantial variability in response to treatment and long-term prognosis that cannot be predicted based on standard histopathology. That 5-year survival rate has improved only marginally over the past several decades. Although treatment paradigms have evolved significantly over time, there has been little change in 5-year survival since the 1970’s. Conventional treatment will usually employ surgery and adjuvant radiation therapy, with or without chemotherapy depending upon pathologic results. Any of these costly therapies can, and do, produce significant morbidities affecting speech, swallowing, and overall quality of life. Despite these interventions, recurrence of the disease is observed in about 50% of patients with high rates of associated mortality.

The identification of genes specifically affected by DNA methylation represent a way forward to the identification of genes with relevance as potential clinical biomarkers. In HNSCC, promoter methylation of tumour suppressor genes appears to be a common mechanism of transcriptional silencing. Numerous studies have identified promoter methylation of CDKN2A (p16), DAP kinase (DAPK), and DNA repair genes MGMT and MLH1 [2, 3]. Based on these and other studies, the identification of epigenetically silenced genes has become an important tool for identifying potential new tumour suppressors and understanding their mechanisms of action.

With the potential for novel gene discovery in mind, our group previously completed a genome-wide scan of aberrant DNA methylation in DNA samples from 118 HNSCCs [4]. We hoped to identify novel genes affected by aberrant DNA methylation that may play a role in head and neck tumourigenesis but have not been previously identified in this disease. From these studies and others, we identified a cluster of novel Krüppel-type zinc finger protein (ZNF) genes located on chromosome 19q13 that are aberrantly hypermethylated with high frequency in all anatomic sub-sites of HNSCC as well as other epithelial tumours [4, 5]. These genes also showed a significant reduction in gene expression in the primary tumour compared to adjacent mucosa. Two interesting examples of epigenetic silencing in primary HNSCC tumours were the Kruppel family zinc finger proteins ZNF132 and ZNF154 which showed both elevated DNA hypermethylation as well as reduced gene expression with high frequency in HNSCC tumours compared to matching adjacent mucosa from the same patient [5].

Here, we examine the expression of these genes in independent patient cohorts of the Cancer Genome Atlas (TCGA). We also describe our attempts to re-express these proteins in HEK293 cells, as well as an HNSCC cell line (UM-SCC-1). Our results shed some light on possible downstream targets of ZNF154 expression, as well as on additional mechanisms of silencing of these proteins by the chromatin regulator TRIM28 in this disease.

## Materials and methods

### Analysis of global gene expression and DNA methylation data from the Cancer Genome Atlas (TCGA)

Global gene expression, DNA methylation, clinical characteristics, and overall survival data, current as of December 2020, for 530 primary HNSCC patients was downloaded from TCGA database [6]. Overall survival was defined as the time between the date of surgery, and the date of death or the last follow-up date. In addition, within the TCGA dataset, gene expression and methylation data for primary tumour and adjacent non-tumor tissue samples were available for 50 primary HNSCC patients. The HNSCC tumours were categorized based on site into three categories—oral cavity (alveolar ridge, buccal mucosa, floor of mouth, hard palate, and oral tongue); oropharynx (base of tongue, uvula, soft palate, and tonsil); and larynx (hypopharynx, and larynx). Tobacco smoking status was classified as ‘Ever smoker’ or ‘Lifelong non-smoker’. In addition to HNSCC, gene expression data was also downloaded for 492 primary lung adenocarcinoma (LUAD) patients from the TCGA.

Data is presented as Mean ± SD, unless otherwise stated. Analyses were conducted using R3.2.1. GraphPad Prism5 (GraphPad, San Diego, CA, USA) and R scripts and Microsoft Excel were used to construct figures. Measurements of gene expression and DNA methylation profiles were represented as continuous variables, whereas clinical data are represented as categorical variables. Comparisons between HNSCC based on primary tumour site were performed using one-way analysis of variance (ANOVA) with post-hoc Bonferroni correction. Comparisons between HNSCC primary tumour and adjacent non-tumor tissue samples were performed using paired t-test, or the Wilcoxon signed-rank test, as appropriate. Overall survival curves for these patient cohorts were assessed using Kaplan–Meier analysis and the log-rank test to assess differences between curves. The association between ZNF154 or ZNF132 gene expression and clinical characteristics were analyzed using the Chi-square test. In all cases, a threshold p-value of *p* < 0.05 was accepted as statistically significant.

### Growth of cell lines and lentiviral transduction of ZNF154 and ZNF132 expressing constructs

Head and neck squamous cell carcinoma (HNSCC) cell line UM-SCC-1 (Cat# SCC070, Millipore Sigma) was maintained at 37 °C, 5%CO_2_. UM-SCC-1 cells were cultured in DMEM/F12 (Cat# SH30023FS, HyClone) supplemented with 10% fetal bovine serum (FBS) (Cat# 081150, Wisent) and non-essential amino acids (Cat# TMS-001-C, Millipore Sigma). Isolation of total RNA was carried out using the RNeasy total RNA kit (Cat# 74104, Qiagen).

Stable constitutive overexpression of ZNF154 and ZNF132 protein in UM-SCC-1 and HEK-293 cells was carried out by lentiviral transduction using the pLenti-C-Myc-DDK-P2A-Puro vector expressing either the tagged ZNF154 or ZNF132 fusion protein under the control of the CMV promoter (Origene Cat# RC219041L3, RC218278L3), followed by selection of transduced clones using 0.25–0.50 μg/mL of puromycin. Transduction using the empty pLenti-C-Myc-DDK-P2A-Puro vector was used as a negative control for each cell type (Origene Cat# PS100092). Expression of ZNF proteins were confirmed at the RNA transcript level by Taqman real-time PCR using the protocol as described by the manufacturer (Thermo Fisher Scientific). Taqman probes for measurement of gene expression were as follows: ZNF154 (Hs06618628_s1) and ZNF132 (Hs01036387_m1). PCR amplification of integrated viral DNA was carried out using internal vector primers V2 (5′-AGAGCTCGTTTAGTGAA-3′) and LR50 (5′-CAGAGGTTGATTATCGATAAG-3′). PCR reactions were carried out using the Phusion Green Hot Start II High Fidelity PCR master mix (Thermo Fisher, Cat#F-566S). PCR parameters included an initial denaturation (98 °C, 30 s), followed by 35 cycles of denaturation (98 °C, 10 s), annealing (56 °C, 30 s) and extension (72 °C, 2 min), with a final extension (72 °C, 10 min). PCR products were then resolved on a 0.8% agarose gel.

For measurements of cell growth, cells were plated and triplicate wells were harvested every 48 h. Cells counts were obtained using a haemocytometer and phase contrast microscope; cell viability was confirmed by Trypan Blue exclusion. All tumor cell numbers were expressed as mean ± SD. Differences between two independent groups were assessed by single factor AVOVA using Microsoft Excel 2013. A p-value of less than 0.05 was considered as the threshold for statistical significance.

### Measurement of FLAG-tagged protein expression

Expression of ZNF154 and ZNF132 protein in lentiviral transductants was determined through quantification of Flag fusion-proteins by Western blot. Transduced cells plated in six well plates were washed with cold phosphate buffered saline and lysed with 200 µl RIPA buffer (50 mM Tris pH7.4, 137 mM NaCl, 2.7 mM KCl, 11.9 mM phosphates, 1% TritonX, 5 mM EDTA, 0.5% deoxycholic acid, 0.1% SDS, 50 mM β-glycerophosphate, 50 mM sodium fluoride, 1 mM PMSF, 2 mM sodium orthovanate, 10 µg/ml Aprotinin, 10 µg/ml Leupeptin and 10 µg/ml Pepstatin) and removed from the plate surface by cell scraper. Lysates were passed through a 25 gauge needle and sonicated three times for 30 s with 30 s incubation intervals on ice. Samples were then centrifuged at 11,000 × g for 15 min at 4 °C to remove cell debris. Protein concentrations were measured using the Pierce BCA protein assay kit (Cat# 2322S, Thermo Fisher Scientific) as per manufacturer’s instructions. Equal amounts of total protein (60 µg) were loaded onto a 10% SDS-PAGE gel, and resolved proteins were transferred onto a nitrocellulose membrane (Cat# 1620115, BioRad). Membranes were probed using a Flag primary antibody (1:2000) (Cat# TA50011, Origene) and secondary antibody (goat anti-mouse HRP (1:5000) (Cat# 115-035-071, Jackson Immunoresearch). HRP signal was detected using Amersham ECL Select Western Blotting Detection Reagent (RPN2235) and imaged on a Biorad Chemidoc MP Imaging System. The membranes were reprobed for β-actin (1:2000) (Cat# MABT523, Millipore Sigma) in the presence of 0.05% sodium azide; secondary antibody was goat anti-rabbit HRP (1:10,000) (Cat# 65-6120, Invitrogen). HRP signal was detected using SuperSignal West Pico PLUS Chemiluminescent Substrate (Cat# 34579, Thermo Fisher Scientific) and imaged on a Biorad Chemidoc MP Imaging System. All images were analyzed using Bio-rad Laboratories Image Lab software (version 6.1).

### Measurement of tumor cell migration

Cell migration was measured using the Radius 96-well cell migration assay (Cat# CBA-126, Cell Biolabs). Culture wells were pre-incubated with gel pre-treatment solution at room temperature for 20 min. Cells were harvested and incubated in assay wells at 37 °C, 5% CO_2_ until gel free area reached 90% confluence. Cells were then starved with serum free media (1% FBS) for an additional 24 h, the gel was removed using gel removal solution. Cells were then washed three times with serum free media (1% FBS). Images of cell migration were captured at 0, 24 and 48 h post gel removal. Cell free area was measured using Adobe Photoshop (version 23.2.2.325).

### Screening of ZNF154 targets using antibody arrays

Changes in response to ZNF154 expression in HEK-293 cells were screened using the Human XL Oncology antibody array according to the manufacturer’s recommended protocol using 200 μg of total protein extract. An integrated density was measured using ImageJ image processing software, and signals were normalized to array reference spots on each array in the experiment. Results were presented as relative expression as compared to the parental HEK-293 cells.

## Results

### Epigenetic downregulation of ZNF154 and ZNF132 expression occurs with high frequency in HNSCC tumours

Our first objective was to validate the previous findings that ZNF154 and ZNF132 were epigenetically silenced in HNSCC tumors, and to test whether their expression might be prognostically relevant in this disease. From the original downloaded data for 530 HNSCC patients, we excluded 11 patients that did not have any clinical data available. We also excluded patients with primary tumours originating from the lip (n = 3), bringing our cohort to 516 HNSCC patients. The characteristics of our overall cohort of 516 HNSCC patients are presented in Table 1. Characteristics of the 50 HNSCC patients with matched primary tumour and adjacent non-tumour tissue samples are presented in Table 2. Unfortunately, ZNF154 and ZNF132 expression data was not available for 30 non-tumour tissue samples, so those samples were excluded from our analysis. An overview of the remaining 20 patients are shown in Additional file 1: Table S3.

**Table 1:** HNSCC patient characteristics by tumor site

	Oral cavity N = 310		Oropharynx N = 82		Larynx N = 124	
	N	%	N	%	N	%
*Gender*						
Male	206	66.5	69	84.1	101	81.5
Female	104	33.5	13	15.9	23	18.5
*Race*						
White	266	85.8	76	92.7	99	79.8
Black or African American	20	6.5	6	7.3	19	15.3
Asian	10	3.2	0	0	1	0.8
American Indian or Alaska Native	1	0.3	0	0	1	0.8
Information not available	13	4.2	0	0	4	3.2
*Ethnicity*						
Hispanic/Latino	16	5.2	3	3.7	5	4
Non-Hispanic/Latino	269	86.8	76	92.7	109	87.9
Information not available	25	8.1	3	3.7	10	8.1
*Smoking*						
Ever smoker	215	69.4	56	68.3	113	91.1
Lifelong non-smoker	86	27.7	25	30.5	8	6.5
Information not available	9	2.9	1	1.2	3	2.4
*HPV status*						
HPV +	31	10	56	68.3	11	8.9
HPV −	278	89.7	26	31.7	112	90.3
Indeterminate	1	0.3	0	0	1	0.8
*Vital status*						
Alive	197	63.5	69	84.1	82	66.1
Deceased	113	36.5	13	15.9	42	33.9
*Nodal status*						
Positive	147	47.4	34	41.5	58	46.8
Negative	119	38.4	16	19.5	42	33.9
Information not available	44	14.2	32	39	24	19.4
*Pathologic tumor stage*						
Stage I	19	6.1	4	4.9	2	1.6
Stage II	56	18.1	10	12.2	12	9.7
Stage III	52	16.8	9	11.0	14	11.3
Stage IV	160	51.6	28	34.1	79	63.7
Information not available	23	7.4	31	37.8	17	13.7

**Table 2:** Patient characteristics for 50 HNSCC patients with adjacent tumor and non-tumor samples

	Oral cavity	Oropharynx	Larynx
	N = 32	N = 2	N = 16
*Gender*			
Male	21	2	15
Female	11	0	1
*Race*			
White	28	2	12
Black or African American	2	0	4
Information not available	2	0	0
*Ethnicity*			
Hispanic/Latino	4	0	4
Non-Hispanic/Latino	25	2	12
Information not available	3	0	0
*HPV status*			
HPV +	6	0	2
HPV −	26	2	14
*Vital status*			
Alive	11	0	7
Deceased	21	2	9
*Pathologic tumor stage*			
Stage II	9	1	1
Stage III	10	1	6
Stage IV	13	0	9

**Fig. 1:**
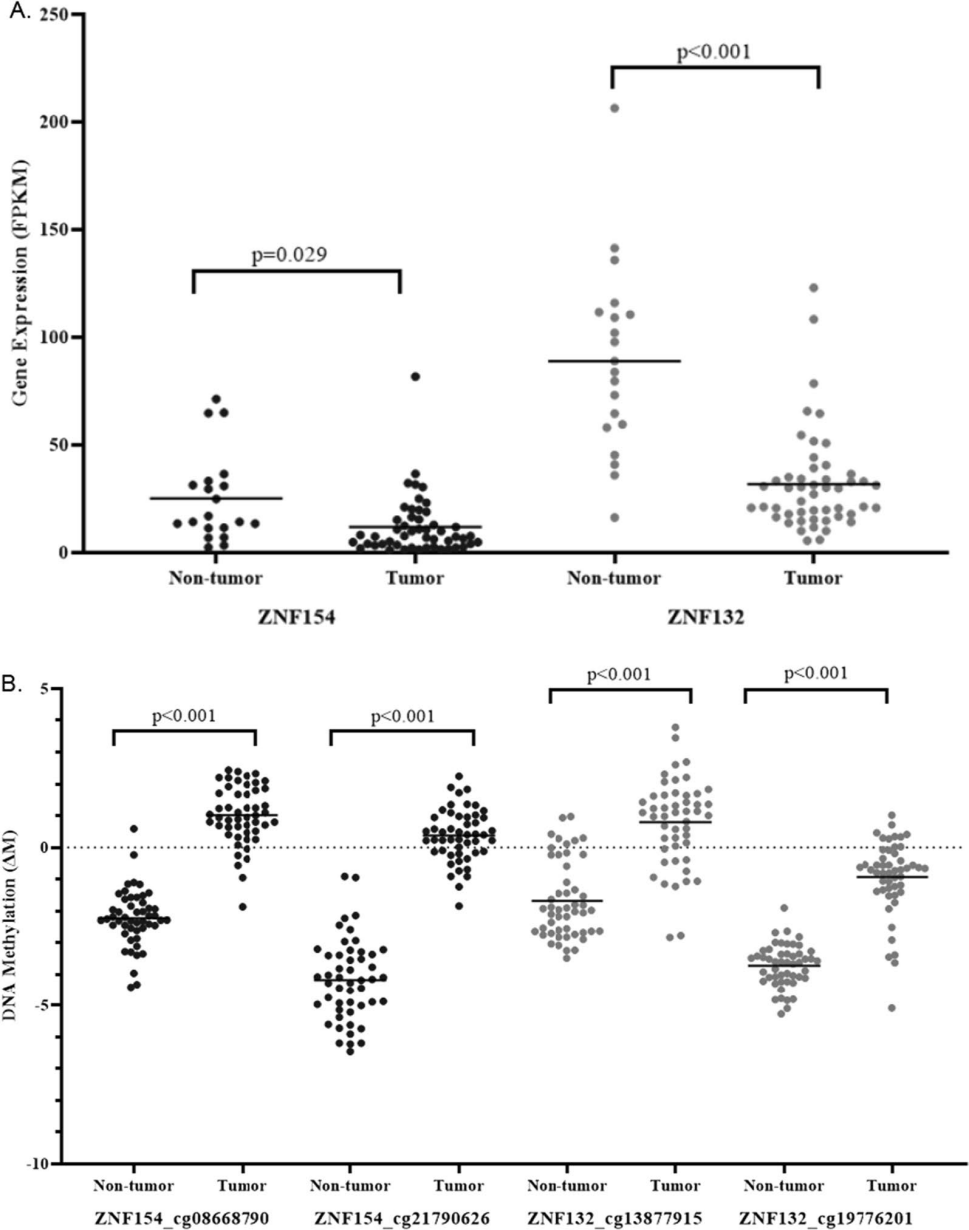
**A** Gene expression and **B** DNA methylation analysis of ZNF154 (black) and ZNF132 (grey) in HNSCC tumor and adjacent non-tumor tissue samples. Specific CpG loci corresponding to each ZNF gene are shown on the x-axis label

RNA sequencing data obtained from the TCGA confirmed that ZNF154 expression was significantly downregulated in HNSCC tumors compared with matching non-tumour tissue from the same patient (tumour 13.80 ± 17.46 versus non-tumour 25.16 ± 20.75, *p* < 0.05) (Fig. 1A). This reduced gene expression in primary tumors was also observed for ZNF132 (tumour 26.38 ± 122.76 versus non-tumour 88.89 ± 43.65, *p* < 0.001). We also obtained data on DNA methylation measurements using the Illumina HumanMethylation450k beadchip. Measurements of DNA methylation (M-values) of two CpG loci located within the ZNF154 promoter CpG island showed significantly increased DNA methylation in HNSCC tumours compared with matching non-tumour tissue from the same patient (cg08668790: 1.02 ± 0.92 (tumor) versus − 2.22 ± 0.90 (non-tumour) *p* < 0.001, and cg21790626: 0.38 ± 0.83 (tumour) versus − 4.18 ± 1.29 (non-tumour) *p* < 0.001) (Fig. 1B). In the case of ZNF132, methylation of two promoter CpG loci also showed significantly increased DNA methylation in HNSCC tumors compared with matching non-tumour tissue from the same patient (cg13877915: 0.80 ± 1.38 (tumour) versus − 1.72 ± 1.20 (non-tumour), *p* < 0.001), and cg19776201: − 0.93 ± 1.18 (tumour) versus − 3.73 ± 0.67 (non-tumour), *p* < 0.001). Taken together, the results confirm our initial findings of epigenetic downregulation of both ZNF132 and ZNF154 in a separate cohort of HNSCC patients.

### Low expression of ZNF154 and ZNF132 proteins are associated with a worse prognosis in both HNSCC and LUAD patient cohorts

**Fig. 2:**
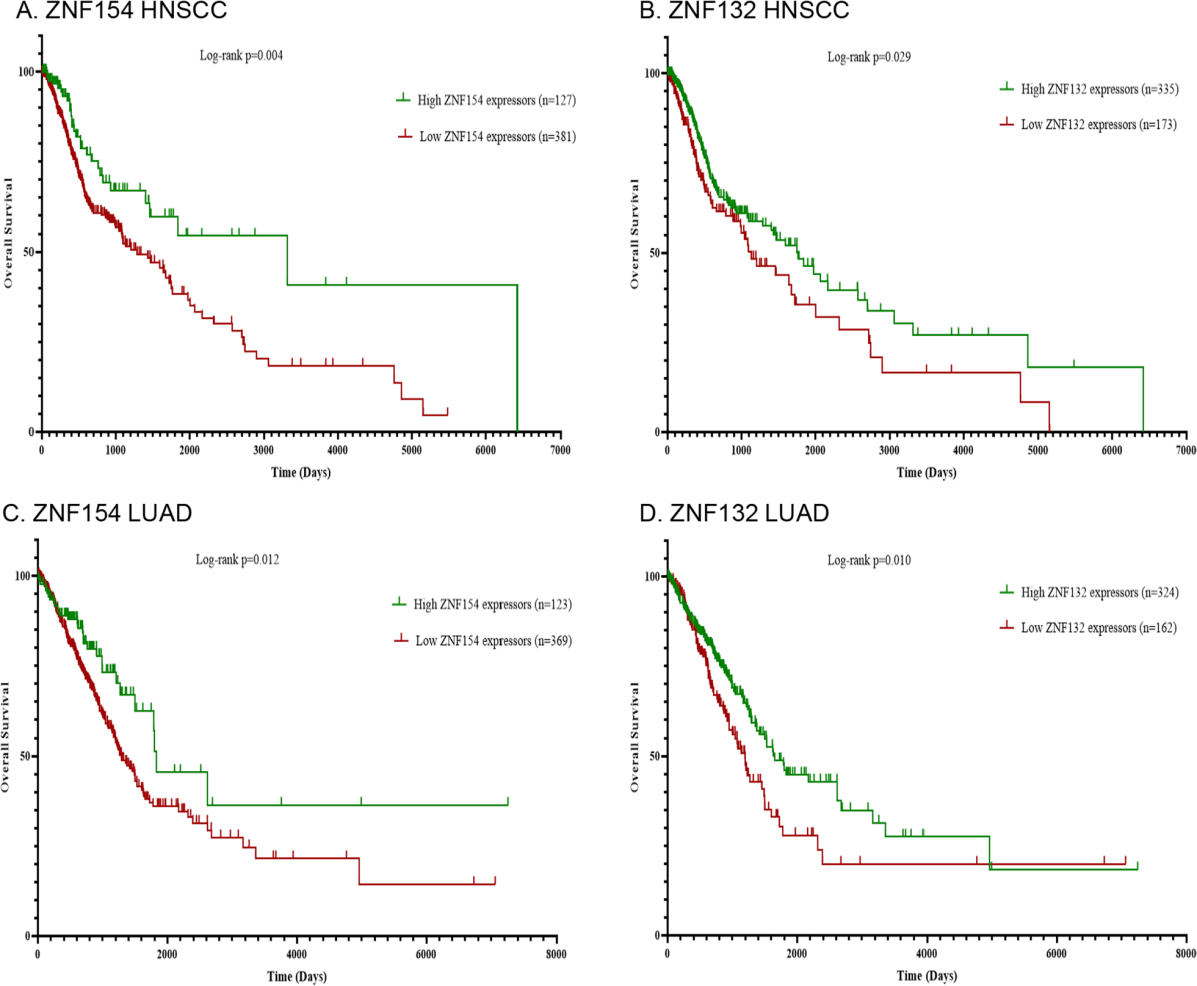
Kaplan–Meier Plots of overall survival (OS) for **A** HNSCC patients stratified by ZNF154 expression, **B** HNSCC patients stratified by ZNF132 expression, **C** LUAD patients stratified by ZNF154 expression, **D** LUAD patients stratified by ZNF132 expression. The red lines indicate low expressors; the green lines indicate high expressors. Difference in survival between patient groups were assessed by Log-rank statistic

Not all HNSCC tumours showed epigenetic silencing of ZNF154 or ZNF132 expression. We therefore tested whether ZNF154 or ZNF132 expression might have prognostic significance in this disease. Utilizing our TCGA cohort of 516 HNSCC patients, we excluded eight HNSCC patients for which gene expression data was not available. In the case of ZNF154, we identified a subgroup of HNSCC patients (n = 127, 25% of cohort) where ZNF154 expression in their primary tumour was not silenced, and was comparable to that observed in non-tumor adjacent tissue. Survival analysis stratifying patients based on this criterion demonstrated that patients whose primary tumours had higher ZNF154 expression showed a significantly better overall survival when compared to the remaining cohort (Fig. 2A, Log-rank, *p* < 0.05). Given the increased range of expression levels for ZNF132 in our patient cohort, we decided to stratify these patients to identify a subgroup with low ZNF132 gene expression (n = 173, 33% of cohort). Survival analysis demonstrated that those HNSCC patients with low ZNF132 expression had a significantly worse overall survival compared to the rest of the patient cohort (Fig. 2B). The patient characteristics of the ZNF154 and ZNF132 subgroupings are shown in Additional file 1: Tables S1 and S2. Expression of ZNF154 was significantly associated with primary tumour site (Chi-square test, *p* < 0.05), while expression of ZNF132 was significantly associated with primary tumour site (Chi-square test, *p* < 0.001), gender (Chi-square test, *p* = 0.016), HPV status (Chi-square test, *p* = 0.005), and pathologic T stage (Chi-square test, *p* = 0.001). There appeared to be a statistically significant association between low ZNF132 expression and HPV positivity. However, the nature of HPV detection in the cohort leaves some uncertainty as to whether this was accompanied by active expression of E6 and E7 oncoproteins. In order to assess the influence of HPV-positivity as a confounding variable, we also removed the HPV DNA positive cases from the cohort and re-assessed the survival analysis using only HPV negative cases. The significant association between high ZNF154 expression and improved overall survival remained; interestingly, the difference in overall survival between ZNF132 low expressors versus the remaining cohort was no longer significant (Additional file 2: Fig. S1). This implies that in the case of ZNF132, HPV-positivity may be a confounding variable in the association between patient survival and ZNF132 expression. The association of high ZNF154 expression with improved overall survival was also validated in a cohort of 53 laryngeal squamous cell carcinoma patients undergoing treatment with curative intent as part of the earlier Albert Einstein College of Medicine Head and Neck Cancer cohort (Log-rank, *p* < 0.05) (Additional file 2: Fig. S2). Unfortunately, we were unable to validate the association of overall survival to ZNF132 expression in this cohort. This may have been due to the much smaller size of this patient cohort when compared to the TCGA.

The prognostic value of ZNF154 and ZNF132 expression was similarly observed in patients from the lung adenocarcinoma (LUAD) TCGA patient cohort. Using the same stratification criteria described above but applied to a cohort of 492 LUAD patients, we found that patients whose primary tumours expressed higher ZNF154 levels (n = 123, 25% of cohort) also had significantly better overall survival compared to the remaining patient cohort (Fig. 2C, Log-rank, *p* < 0.05). And in the case of ZNF132, patients whose primary tumours expressed low levels of ZNF132 (n = 162, 33%) showed a significantly decreased overall survival when compared to the remaining cohort (Fig. 2D, Log-rank, *p* < 0.05). These observations were consistent with those of the HNSCC patient cohort, and support a prognostic role for ZNF154 and ZNF132 expression as a possible prognostic biomarker in both lung and upper aerodigestive malignancies.

### Overexpression of ZNF154 and ZNF132 proteins in HEK-293 and head and neck squamous cell carcinoma cell line UM-SCC-1

To investigate the possible tumour suppressive properties of ZNF154 and ZNF132 proteins in head and neck cancer, we attempted to overexpress these proteins in a head and neck squamous cell carcinoma cell line (UM-SCC-1) by lentiviral transduction of a C-terminal Flag-tagged fusion protein construct under the control of a CMV promoter. As a control to confirm functionality of the vector constructs for stable overexpression, we also overexpressed both ZNF fusion proteins using the same lentiviral constructs in the human embryonic kidney 293 cell line (HEK-293).

**Fig. 3:**
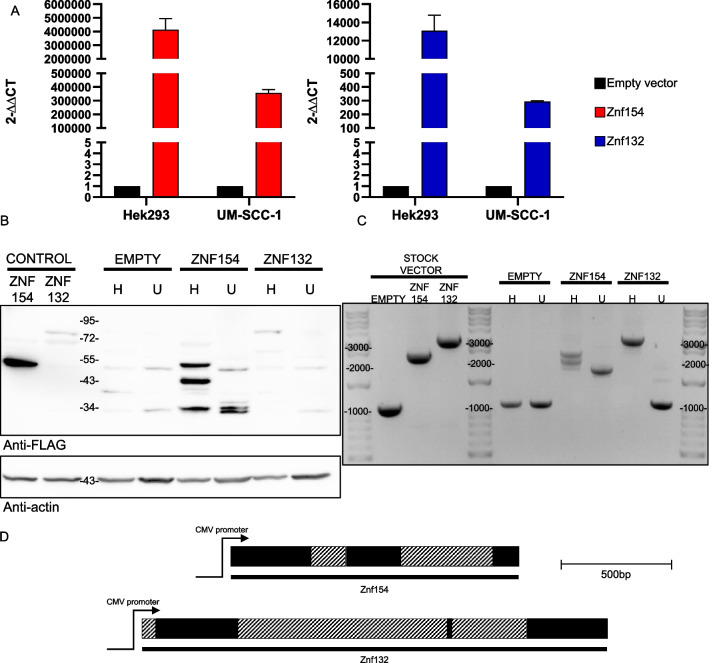
**A** Assessment of ZNF154 and ZNF132 transcripts in HEK-293 and UM-SCC-1 transductant cells and empty vector controls by real-time PCR. RNA transcript measurements are expressed as ΔΔCt values using GAPDH as a measurement control. **B** Western blot assessment of the same lentiviral transductants overexpressing ZNF154 or ZNF132 as probed using a Flag antibody. Also shown are empty vector controls for HEK-293 (H) and UM-SCC-1 (U). β-actin is used as a loading control. **C** PCR amplification of integrated vector DNA constructs for ZNF154-Flag, ZNF132-Flag, and empty vector control in both HEK-293 (H) and UM-SCC-1 cells (U). Stock vector DNA and a water blank were used as PCR positive and negative controls, respectively. **D** Schematic diagram showing locations of deleted internal coding sequences for each ZNF gene in UM-SCC-1 transductants as determined by sequencing of genomic DNA PCR products

We initially confirmed overexpression of the Flag-tagged ZNF154 and ZNF132 fusion-constructs by both real-time quantitative PCR (qPCR) and at the protein level by Western blot. In HEK-293 cells, quantitation of RNA transcripts by Taqman quantitative real-time PCR (qPCR) revealed significant increase in transcript abundance for ZNF154 and ZNF132 in the transduced HEK-293 clones when compared to the empty vector control (Fig. 3A). Similarly, significant expression of both ZNF transcripts were observed in UM-SCC-1 cancer cells that were both undetectable in empty vector UM-SCC-1 control cells. At the protein level, Western blot analysis demonstrated a similar over-expression of both Flag-tagged ZNF proteins in HEK-293 cells, with molecular weights of 50 kDa (ZNF154) and 82 kDa (ZNF132) (Fig. 3B). These proteins were absent in the empty vector control cells. While overexpression of Flag-tagged fusion proteins were observed in UM-SCC-1 cancer cells, both proteins were smaller in size then the expected molecular weights, with molecular weights of approximately 34 kDa in both transductants.

In order to establish the basis for the reduced protein sizes at the gene level, we checked whether complete ZNF gene sequences were successfully integrated into the host genomic DNA. PCR amplification using primers internal to the vector sequence at each end of the ZNF gene insert should produce PCR products of 2340 bp (ZNF154), 3147 bp (ZNF132) and 1067 bp (empty vector control). PCR amplification from host cell genomic DNA showed that all lentiviral transduction of HEK-293 cells resulted in stably integrated DNA sequences corresponding to the expected sizes of 2340 bp (ZNF154), 3147 bp (ZNF132) and 1067 bp (empty vector control) (Fig. 3C). In contrast, transduction of the same constructs into UM-SCC-1 cells resulted in stably integrated DNA sequences of a length that was shorter than expected. All empty vector controls produced PCR products of the expected size (1067 bp). This same trend was observed in all isolated clones. Sequencing of these truncated PCR products from UM-SCC-1 cells for each construct revealed a deletion of internal coding sequences for both ZNF genes (Fig. 3D). From this, we suspect that other regulatory mechanisms, in addition to aberrant promoter DNA hypermethylation, may play a role in the transcriptional silencing of these genes in oral cancer cells that are not a factor in HEK-293 cells.

### Overexpression of partial ZNF154 construct affects HNSCC tumor cell phenotypes

**Fig. 4:**
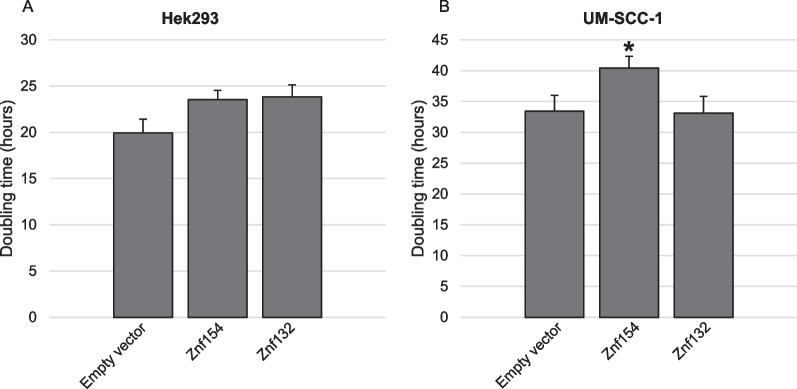
**A** Comparison of doubling times for HEK-293 transductants expressing full length ZNF154, ZNF132 or the empty vector control. **B** Comparison of doubling times for UM-SCC-1 transductants expressing partial length ZNF154, ZNF132 or the empty vector control

In spite of the fact that the HNSCC cell line produced truncated ZNF154 and ZNF132 proteins, we wanted to examine what effects these partial proteins might have on tumor cell phenotype. We also wanted to test any effects of the full-length ZNF proteins on HEK-293 growth. In the case of HEK-293 cells, we measured growth of cells overexpressing either the ZNF154 or ZNF132 protein and compared each to cells containing the empty vector construct. In both cases, overexpression of either the ZNF154 protein or ZNF132 protein resulted in a slight increase in doubling time (23.5 and 23.8 h, respectively) when compared to the empty vector control (19.9 h) (Fig. 4A). For comparison, we also measured growth of UM-SCC-1 cells expressing the truncated ZNF154 and ZNF132 proteins (Fig. 4B). In this case, expression of the truncated ZNF154 construct resulted in a significant increase in UM-SCC-1 doubling time (40.4 h) when compared to the empty vector control (33.4 h). This reduction was not observed for cells overexpressing the ZNF132 partial construct (33.1 h). From these results, we concluded that even when expressed as a partial sequence, ZNF154 peptide reduced doubling time for UM-SCC-1 cancer cells while neither full length ZNF protein had significant effect on growth of HEK-293 cells.

**Fig. 5:**
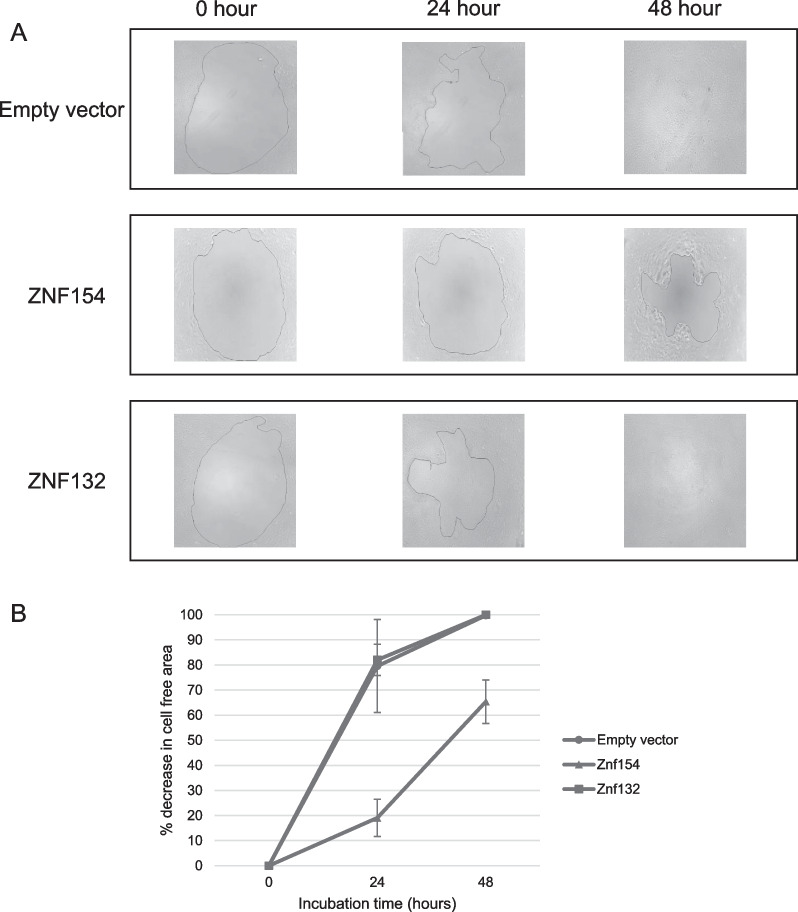
**A** Representative images of wound healing assay comparing cell mobility of HNSCC cancer UM-SCC-1 cells containing empty vector, ZNF154 construct, or ZNF132 construct. Images were taken at 0, 24 and 48 h. **B** Mobility was quantified as a reduction in the percentage of cell free area after 24 and 48 h

In addition to growth, migration of UM-SCC-1 cancer cells was measured using the Radius 96-well cell migration assay. As with the growth assay, overexpression of the truncated ZNF154 protein resulted in a significant decrease in tumor cell migration when compared to the empty vector control cells (Fig. 5A, B). However, this decrease in cell migration was not observed in UM-SCC-1 cells overexpressing the truncated ZNF132 protein. From these and the previous results, it appeared that even the truncated version of ZNF154 retained some biological activity resulting in the reduction in both growth and tumor cell migration. However, the truncated version of ZNF132 appeared to have neither a growth of migratory effect in vitro, a result inconsistent with the survival associations in Fig. 2.

**Fig. 6:**
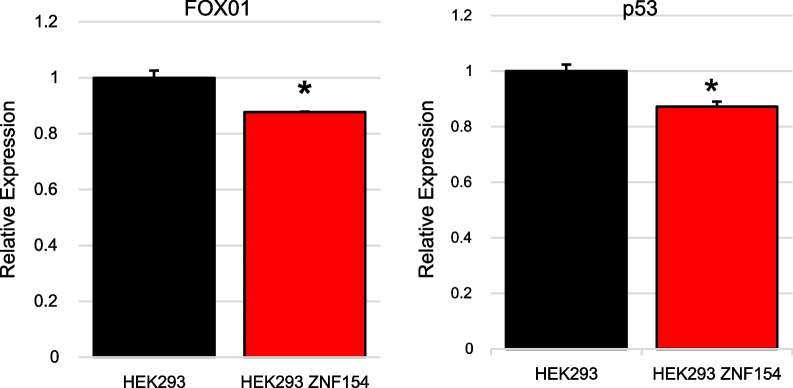
Measurement of FOXO1 and P53 protein as determined by the antibody array in HEK293 cells overexpressing ZNF154 compared to the HEK293 empty vector control

We wanted to test whether we could identify potential downstream targets of ZNF154 in its full-length form. To do this, we compared total protein extracts from parental HEK-293 cells to those overexpressing the full-length ZNF154 protein using the Proteome Profiler Human XL Oncology Antibody Array. This array compares relative expression levels of 84 human cancer-related proteins in a single experiment (Fig. 6). Comparisons of protein extracts revealed a significant reduction in both the levels of p53 and Forkhead box protein FOXO1 in cells overexpressing ZNF154 protein compared to the parent HEK-293 cells. However, the relevance of these changes to head and neck cancer cells is still not clear.

## Discussion

Inactivation of tumour suppressor genes due to promoter DNA hypermethylation is thought to be one of the most common transcriptional gene silencing mechanisms in human malignancies. In this current study, we found in genomic datasets obtained from the TCGA that both ZNF154 and ZNF132 were silenced and hypermethylated in a significant number of HNSCC primary tumour samples when compared to adjacent non-tumor samples from the same patient. Our research group previously identified six novel KRAB-ZNF genes (ZNF154, ZNF132, ZNF542, ZNF545/ZFP82 and ZNF781) that were hypermethylated with high frequency in HNSCC tissue samples [4]. ZNF154 and ZNF132 were also observed to be hypermethylated in a previous dataset of oral cavity SCC cases [7].

The epigenetic silencing of ZNF154 and ZNF132 were not confined to head and neck cancers. In 2013, Sánchez-Vega and coworkers found ZNF154 hypermethylation and downregulation to be one of the most common epigenetic changes in ovarian and endometrial cancers, and subsequently, across 15 distinct solid cancer types from the TCGA [8]. The importance of ZNF154 in cancer is further highlighted by recurring reports of its hypermethylation in several other malignancies, including bladder [9], breast [10], lung [11], ovarian [12], renal [13] and prostate [14]. Hypermethylation of ZNF154 has also been identified in hepatocellular carcinoma, and as part of a panel of early detection biomarkers in DNA from voided urine of bladder cancer patients [9, 15]. ZNF154 methylation has most recently been documented in plasma samples from early-stage cancer patients, suggesting it as a promising target in liquid biopsy [16]. That same study demonstrated that ZNF154 circulating-free DNA (cfDNA) methylation discriminated cases from healthy donor plasma samples and outperformed KRAS mutation frequency as a biomarker in pancreatic cancer. In the case of ZNF132, it was observed to be downregulated in prostate cancer [17], breast cancer [18] and esophageal squamous cell carcinomas [19].

Epigenetic regulation of ZNF154 and ZNF132 may also have prognostic relevance for both HNSCC and LUAD patients. Consistent with their roles as tumor suppressors, we found that low expression of either ZNF154 or ZNF132 were significantly associated with a worse overall survival in 508 HNSCC patients. Those associations were also observed in 492 LUAD patients with gene expression data from the TCGA database. Consistent with our observations, Kaplan–Meier analysis also showed that hypermethylation of the ZNF154 promoter was associated with significantly poorer disease-free survival (*p* = 0.032) and distant metastasis-free survival (*p* = 0.040) among patients with locoregionally advanced nasopharyngeal carcinoma (NPC) [20]. Kaplan–Meier analysis has also shown that ZNF154 methylation level was associated with biochemical recurrence (BCR) (*p* = 0.005) in prostate cancer, and that ZNF154 could be an independent factor for BCR prediction by using univariate and multivariate Cox regression analysis (*p* = 0.035, HR = 8.218) [21]. This association was observed despite the fact that these cancers represent differing cancer biologies. Curiously, an opposite association was observed in pancreatic cancer patients who had undergone a pancreatic resection, where a silenced ZNF154 gene was actually associated with a better patient survival [22]. The reason for this is unclear, but the investigators suggest that silencing of ZNF154 might foster the growth of more stable, less aggressive tumor clones [22]. In the case of ZNF132, low protein expression was associated with a higher Gleason score and advanced T stage in prostate cancer patients, indicating more a aggressive and progressive disease phenotype [17].

While we were able to express full-length ZNF154 and ZNF132 constructs in HEK-293 cells without issue, attempts to express these same constructs in head and neck UM-SCC-1 cancer cells resulted in truncated proteins lacking partial internal amino acid sequences. In another HNSCC cell line (SCC-25), similarly transduced cells showed no expression of either ZNF154 or ZNF132 despite their selection for puromycin-resistance (data not shown). One explanation for this is the observation that global chromatin regulator TRIM28 can suppress transcription of some ZNF genes via binding to the internal coding sequence of the gene, in a mechanism that itself utilizes other ZNF proteins [23]. The absence of internal sequences within the ZNF154 and ZNF132 constructs of UM-SCC-1 supports this as a possible mechanism of silencing that is in addition to promoter DNA hypermethylation. However, this mechanism is poorly understood, and we have not yet identified a ZNF protein that may involved in this specific silencing mechanism. Moreover, it is unclear why this mechanism differs between HEK-293 and UM-SCC-1 cells. Experiments to knockdown TRIM28 expression by siRNA, or inhibit DNA methylation using 5-aza-cytidine, seemed to have little effect on ZNF154 or ZNF132 re-expression in parental UM-SCC-1 cells (data not shown).

In HEK-293 cells, overexpression of ZNF154 resulted in significant downregulation of tumor protein p53 and Forkhead box protein FOXO1. FOXO1 has previously been identified as a possible tumour suppressor in prostate cancer cells and glioma cells by upregulating proapoptotic factors [24]. In keratinocytes, nuclear localization of FOXO1 has been shown to enhance the wound healing process by encouraging migration of keratinocytes [25]. Other studies with hypopharynx cancers suggest that ZNF154 has tumour-suppressive action by inhibiting the Wnt/β-catenin signalling pathway activation and suppressing epithelial-mesenchymal transition [20]. And most recently, expression of ZNF154 in MGC-803 gastric cancer cells reduced cell proliferation, viability, migration and invasion, and enhanced cell apoptosis and arrested cell cycle in G2 phase [26]. This overexpression of ZNF154 was associated with an increase in the expression of B-cell lymphoma-2 (Bcl-2), matrix metalloproteinase 1 (MMP-1), hepatocyte growth factor (HGF), vascular endothelial growth factor-A/C (VEGF-A/C). Little is known about potential targets of ZNF132. However, in analysis of breast cancer datasets, ZNF132 has been identified by computational approaches as a potential transcriptional master regulator of several transcriptional processes that are well-known hallmarks of cancer [27].

The study as described suffers from several limitations. First, it was not possible to express a full length ZNF154 or ZNF132 construct within oral cancer UM-SCC-1 cells in order to identify possible downstream targets of these proteins in the cancer cell environment. This is despite the fact that the expression system is fully functional within HEK-293 cells. Moreover, our observations are derived from a single HNSCC cell line. UM-SCC-1 cells were the only cell line identified to date that would produce ZNF154 or ZNF132, albeit in a truncated form. We hypothesize that the global chromatin regulator TRIM28 can suppress transcription of ZNF154 and ZNF132 via binding to the internal coding sequence of each gene. Expression was not possible in SCC-25 or SCC-15 oral cancer cells despite several attempts (data not shown). We continue to screen HNSCC cell lines to identify those that might express these as full length proteins. We have expressed another KRAB-ZNF protein (ZNF671) in UM-SCC-1 cells, but also found it was not possible to express ZNF671 protein within other oral cancer cell lines such as SCC-25 and SCC-15 (data not shown). The exact mechanisms responsible for this selective expression is unknown but likely will have significance in head and neck and other cancers. It is also not known what effect these proteins would have on tumor cell phenotype in vivo. However, a recent support supporting a tumour suppressive role for ZNF154 included data showing that targeted expression of ZNF154 inhibited expression of esophageal squamous cell carcinoma cells in vivo [28]. Our future studies include plans to evaluate in vivo effects of ZNF overexpression in a floor of mouth mouse model.

In conclusion, aberrant hypermethylation of ZNF154 and ZNF132 mediated their silencing in primary HNSCC tumor tissue samples. Low ZNF154 and low ZNF132 expression were associated with shorter overall survival in both HNSCC and LUAD patients. Future studies are needed to address the underlying molecular mechanisms regulating ZNF154 and ZNF132 expression in HNSCC and other malignancies, their potential as diagnostic and prognostic markers, and the downstream genes that are possible targets for their suppression.

## Supplementary Information

**Additional file 1: Table S1.** Clinical association between ZNF154 expression and clinicopathological variables. **Table S2.** Clinical association between ZNF132 expression and clinicopathological variables. **Table S3.** Patient characteristics for 20 HNSCC patients with adjacent tumor and non-tumor samples. **Table S4.** Summary of 53 larynx squamous cell carcinoma cases obtained from the Albert Einstein College of Medicine Head and Neck Cancer Database.

**Additional file 2: Figure S1.** Kaplan–Meier Plots of overall survivalfor **A** HPV-negative HNSCC patients stratified by ZNF154 expression, **B** HPV-negative HNSCC patients stratified by ZNF132 expression. The red lines indicate low expressors; the green lines indicate high expressors. Difference in survival between patient groups were assessed by Log-rank statistic. **Figure S2.** Kaplan–Meier Plots of overall survivalfor 53 larynx cancer cases derived from the Albert Einstein College of Medicine Head and Neck Cancer database. Stratified by ZNF154 expression, HPV-negative HNSCC patients stratified by ZNF132 expression. The red line indicate low ZNF154 expressors; the teal line indicate high ZNF154 expressors. Difference in survival between patient groups was assessed by Log-rank statistic.

## Data Availability

The datasets analysed during the current study are available in the TCGA repository, (https://www.cbioportal.org/study/summary?id=hnsc_tcga) [6].

## Abbreviations

ANOVA: Analysis of variance
BCR: Biochemical recurrence
Bcl-2: B-cell lymphoma-2
cfDNA: Circulating free DNA
CMV: Cytomegalovirus
DAPK: DAP kinase
DNA: Deoxyribonucleic acid
FBS: Fetal bovine serum
HEK-293: Human embryonic kidney-293
HGF: Hepatocyte growth factor
HNSCC: Head and neck squamous cell carcinoma
HPV: Human papillomavirus
HRP: Horse radish peroxidase
KCl: Potassium chloride
KRAB-ZNF: Krüppel-type zinc finger protein
LUAD: Lung adenocarcinoma
MMP: Matrix metalloproteinase
NaCl: Sodium chloride
PCR: Polymerase chain reaction
RNA: Ribonucleic acid
SD: Standard deviation
TCGA: The Cancer Genome Atlas
