# Supplementary material for: Kruppel-family zinc finger proteins as emerging epigenetic biomarkers in head and neck squamous cell carcinoma
Source: J Otolaryngol Head Neck Surg. 2023 May 30;52:41. doi: 10.1186/s40463-023-00640-x (PMC10228066; doi:10.1186/s40463-023-00640-x)
Supplement: Supplementary file 1 — Additional file 1: Table S1. Clinical association between ZNF154 expression and clinicopathological variables. Table S2. Clinical association between ZNF132 expression and clinicopathological variables. Table S3. Patient characteristics for 20 HNSCC patients with adjacent tumor and non-tumor samples. Table S4. Summary of 53 larynx squamous cell carcinoma cases obtained from the Albert Einstein College of Medicine Head and Neck Cancer Database. [file 40463_2023_640_MOESM1_ESM.pdf]

Supplementary Table 1. Clinical association between ZNF154 expression and clinicopathological variables for

	ZNF154 Expression		χ2 test p-value	Correlation	
	High expression (N=127)	Low expression (N=381)		r	p-value
Site			**0.003**	0.148	**0.001**
Oral Cavity	60	245			
Oropharynx	25	55			
Larynx/Hypoharynx	42	81			
Gender			0.907	-0.005	0.908
Male	94	280			
Female	33	101			
Race			0.457	0.016	0.712
White	106	327			
Black or African American	14	31			
Asian	1	10			
American Indian or Alaska Native	0	2			
Information not available	6	11			
Ethnicity			0.312	0.068	0.127
Hispanic/Latino	5	18			
Non-Hispanic/Latino	108	339			
Information not available	14	24			
Smoking			0.358	0.005	0.911
Ever smoker	99	283			
Lifelong non-smoker	27	87			
Information not available	1	11			
HPV Status			0.696	0.005	0.917
HPV +	25	71			
HPV -	102	308			
Indeterminate	0	2			
Vital Status			**0.004**	0.129	**0.004**
Alive	99	244			
Deceased	28	137			
Nodal Status			0.697	-0.017	0.696
Positive	62	173			
Negative	43	130			
Information not available	22	78			
Pathologic T Stage			0.337	0.027	0.540
T0	0	1			
T1	15	30			
T2	34	99			
T3	18	79			
T4	45	125			
TX	12	26			
Information not available	3	21			
Pathologic N Stage			0.294	-0.026	0.561
N0	43	130			
N1	20	44			
N2	40	123			
N3	2	6			
Information not available	22	78			
Pathologic M Stage			0.942	0.002	0.970
M0	44	133			
M1	0	1			
MX	15	42			
Information not available	68	205			
Pathologic Tumor Stage			0.969	0.018	0.685
Stage I	6	19			
Stage II	17	58			
Stage III	20	54			
Stage IV	67	196			
Information not available	17	54			

Significant differences are bolded.

**Supplementary Table 2. Clinical association between ZNF132 expression and clinicopathological variables for**					
	ZNF132 Expression		χ2 test p-value	Correlation	
	High expression (N=335)	Low expression (N=173)		r	p-value
Site			**<0.001**	0.162	**<0.001**
Oral Cavity	175	130			
Oropharynx	72	8			
Larynx/Hypoharynx	88	35			
Gender			**0.016**	-0.107	**0.016**
Male	258	116			
Female	77	57			
Race			0.468	0.017	0.694
White	282	151			
Black or African American	33	12			
Asian	8	3			
American Indian or Alaska Native	1	1			
Information not available	11	6			
Ethnicity			0.462	-0.017	0.700
Hispanic/Latino	13	10			
Non-Hispanic/Latino	297	150			
Information not available	25	13			
Smoking			0.162	-0.084	**0.047**
Ever smoker	259	123			
Lifelong non-smoker	67	47			
Information not available	9	3			
HPV Status			**0.005**	0.143	**0.001**
HPV +	76	20			
HPV -	257	153			
Indeterminate	2	0			
Vital Status			**0.032**	0.096	**0.031**
Alive	237	106			
Deceased	98	67			
Nodal Status			0.706	0.035	0.425
Positive	157	78			
Negative	110	63			
Information not available	68	32			
Pathologic T Stage			**0.001**	-0.083	**0.060**
T0	1	0			
T1	40	5			
T2	88	45			
T3	63	34			
T4	97	69			
TX	30	8			
Information not available	16	8			
Pathologic N Stage			0.744	0.049	0.276
N0	110	63			
N1	38	26			
N2	112	51			
N3	7	1			
Information not available	68	32			
Pathologic M Stage			0.138	0.018	0.681
M0	112	65			
M1	1	0			
MX	45	12			
Information not available	177	96			
Pathologic Tumor Stage			0.122	-0.105	**0.018**
Stage I	22	3			
Stage II	48	27			
Stage III	47	27			
Stage IV	165	98			
Information not available	53	18			
Significant differences are bolded.					

Supplementary Table 3. Patient characteristics for 20 HNSCC patients with adjacent tumor and non-tumor samples

	Oral Cavity N=14	Oropharynx N=1	Larynx N=5
**Gender**			
Male	9	1	5
Female	5	0	0
**Race**			
White	13	1	5
Black or African American	0	0	0
Information not available	1	0	0
**Ethnicity**			
Hispanic/Latino	1	0	2
Non-Hispanic/Latino	12	1	3
Information not available	1	0	0
**HPV Status**			
HPV +	3	0	1
HPV -	11	1	4
**Vital Status**			
Alive	2	0	1
Deceased	12	1	4
**Pathologic Tumor Stage**			
Stage II	6	0	1
Stage III	4	1	1
Stage IV	4	0	3

Supplementary Table 4. Summary of 53 Larynx Squamous Cell Carcinoma Cases obtained from the Albert Einstein College of Medicine Head and Neck Cancer Database.

	**Overall**
**N**	**53**
age (mean (SD))	63.34 (11.74)
gender = M (%)	34 (64.2)
race = White (%)	36 (67.9)
ethnicity = non-Hispanic/Latino (%)	41 (77.4)
**tumor_site (%)**	
HYPOPHARYNX - pyriform sinus	1 (1.9)
LARYNX-SUPRAGLOTTIS - epiglottis	6 (11.3)
LARYNX-SUPRAGLOTTIS - NOS	31 (58.5)
LARYNX - glottis (true vocal cord)	15 (28.3)
**final_t_stage (%)**	
1	4 (7.5)
2	10 (18.9)
3	27 (50.9)
4	1 (1.9)
4a	10 (18.9)
N.D.	1 (1.9)
**final_n_stage (%)**	
0	28 (52.8)
1	5 (9.4)
2a	1 (1.9)
2b	5 (9.4)
2c	13 (24.5)
N.D.	1 (1.9)
**final_m_stage (%)**	
0	51 (96.2)
1	1 (1.9)
N.D.	1 (1.9)
**final_stage (%)**	
I	2 (3.8)
II	7 (13.2)
III	17 (32.1)
IVa	25 (47.2)
IVc	1 (1.9)
N.D.	1 (1.9)
**ZNF154_status = Low (%)**	**30 (56.6)**
