# Supplementary material for: Kruppel-family zinc finger proteins as emerging epigenetic biomarkers in head and neck squamous cell carcinoma
Source: J Otolaryngol Head Neck Surg. 2023 May 30;52:41. doi: 10.1186/s40463-023-00640-x (PMC10228066; doi:10.1186/s40463-023-00640-x)
Supplement: Supplementary file 2 — Additional file 2: Figure S1. Kaplan–Meier Plots of overall survivalfor A HPV-negative HNSCC patients stratified by ZNF154 expression, B HPV-negative HNSCC patients stratified by ZNF132 expression. The red lines indicate low expressors; the green lines indicate high expressors. Difference in survival between patient groups were assessed by Log-rank statistic. Figure S2. Kaplan–Meier Plots of overall survivalfor 53 larynx cancer cases derived from the Albert Einstein College of Medicine Head and Neck Cancer database. Stratified by ZNF154 expression, HPV-negative HNSCC patients stratified by ZNF132 expression. The red line indicate low ZNF154 expressors; the teal line indicate high ZNF154 expressors. Difference in survival between patient groups was assessed by Log-rank statistic. [file 40463_2023_640_MOESM2_ESM.pptx]

## Slide 1
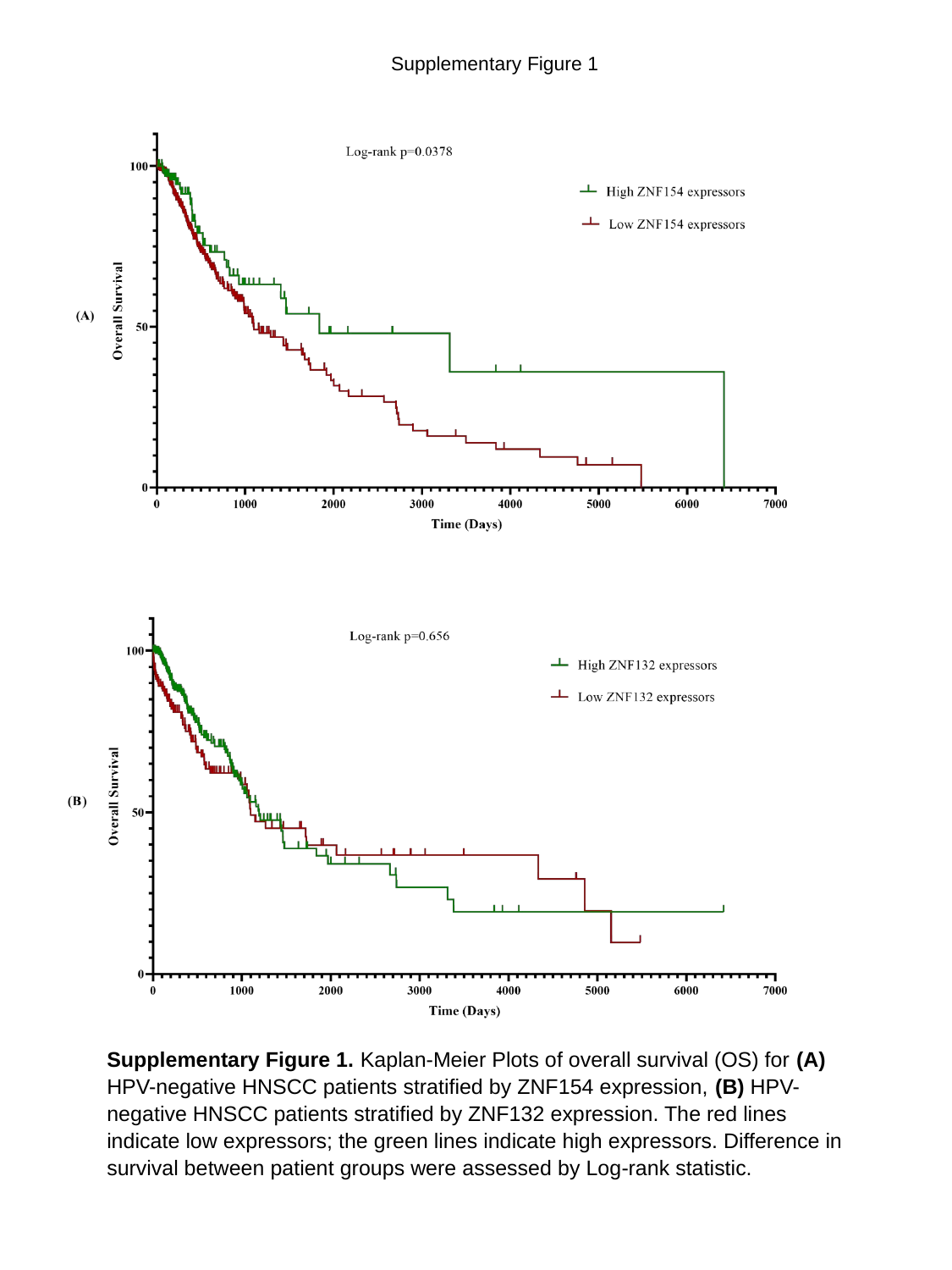

Supplementary Figure 1
Supplementary Figure 1. Kaplan-Meier Plots of overall survival (OS) for (A) HPV-negative HNSCC patients stratified by ZNF154 expression, (B) HPV-negative HNSCC patients stratified by ZNF132 expression. The red lines indicate low expressors; the green lines indicate high expressors. Difference in survival between patient groups were assessed by Log-rank statistic.

## Slide 2
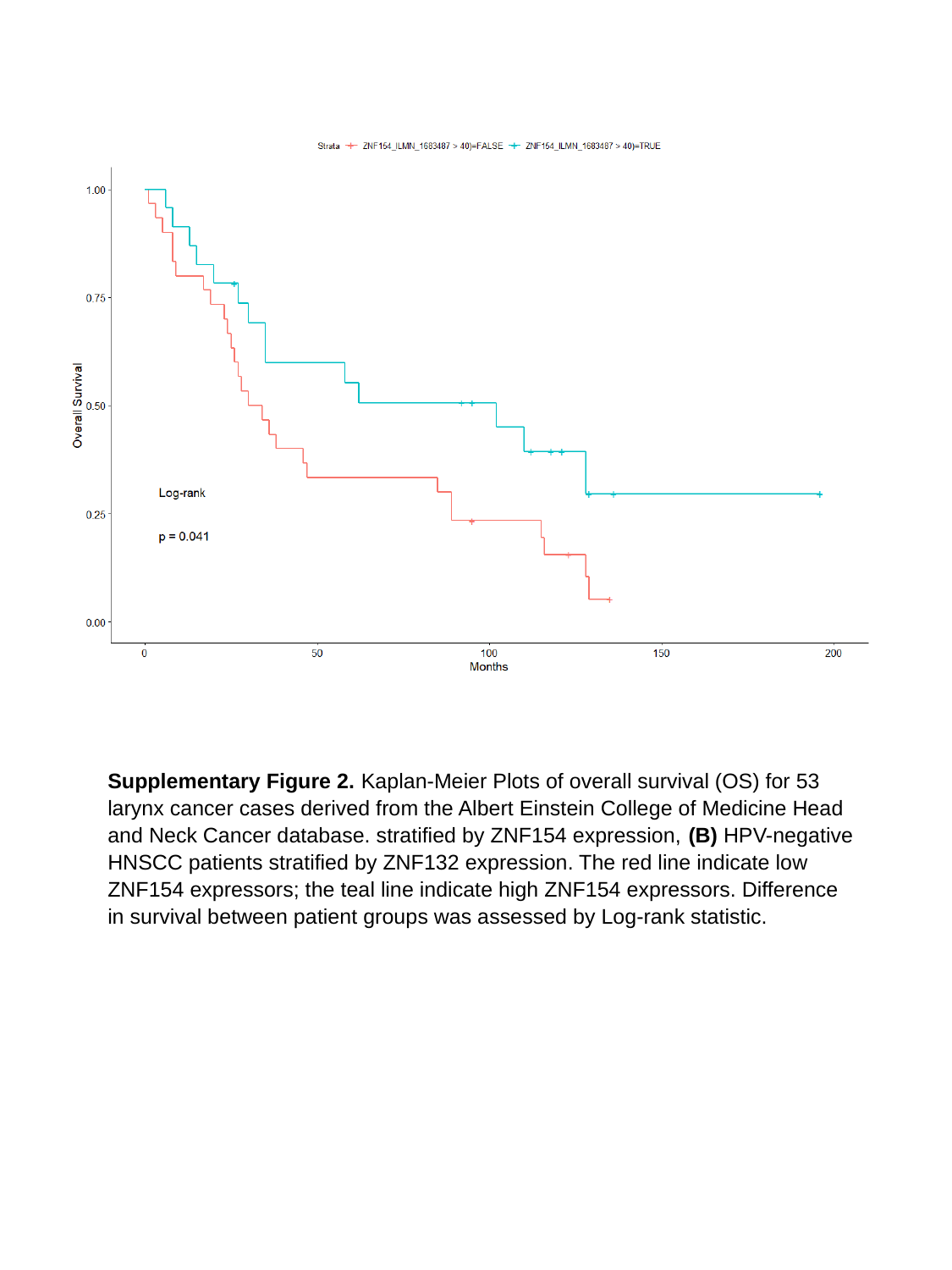

Supplementary Figure 2. Kaplan-Meier Plots of overall survival (OS) for 53 larynx cancer cases derived from the Albert Einstein College of Medicine Head and Neck Cancer database. stratified by ZNF154 expression, (B) HPV-negative HNSCC patients stratified by ZNF132 expression. The red line indicate low ZNF154 expressors; the teal line indicate high ZNF154 expressors. Difference in survival between patient groups was assessed by Log-rank statistic.
